# Supplementary material for: PTRAMP, CSS and Ripr form a conserved complex required for merozoite invasion of Plasmodium species into erythrocytes
Source: Nat Commun. 2026 Jan 26;17:1780. doi: 10.1038/s41467-026-68486-1 (PMC12916814; doi:10.1038/s41467-026-68486-1)
Supplement: Supplementary file 1 — Supplementary Information [file 41467_2026_68486_MOESM1_ESM.pdf]

Supplementary information for

PTRAMP, CSS and Ripr form a conserved complex required for merozoite invasion of *Plasmodium* species into erythrocytes

Seager *et al.*

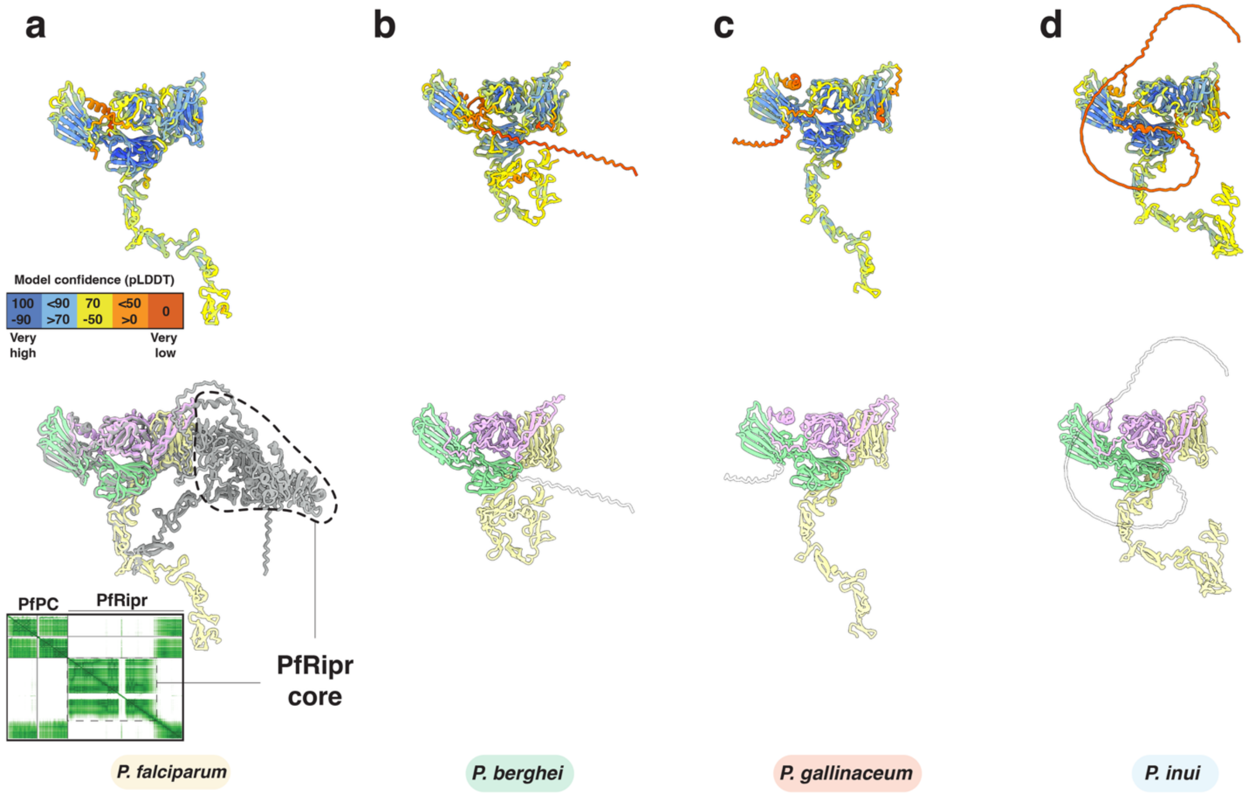

Supplementary Figure 1. AlphaFold 3 predicts a PTRAMP, CSS, Ripr complex for all major clades of *Plasmodium*. a-d. AlphaFold 3 predictions of PTRAMP, CSS and Ripr from several species of *Plasmodium*. Top row: Models colored by confidence (pLDDT). Bottom row: models colored as follows: PTRAMP (pink), CSS (green) and Ripr (yellow). The prediction for *P. falciparum* shows the alignment with full length Ripr, where the core forms an ordered structure. Predicted alignment error (PAE) plot is shown as inset. All models have transmembrane domains and signal peptides omitted for clarity.

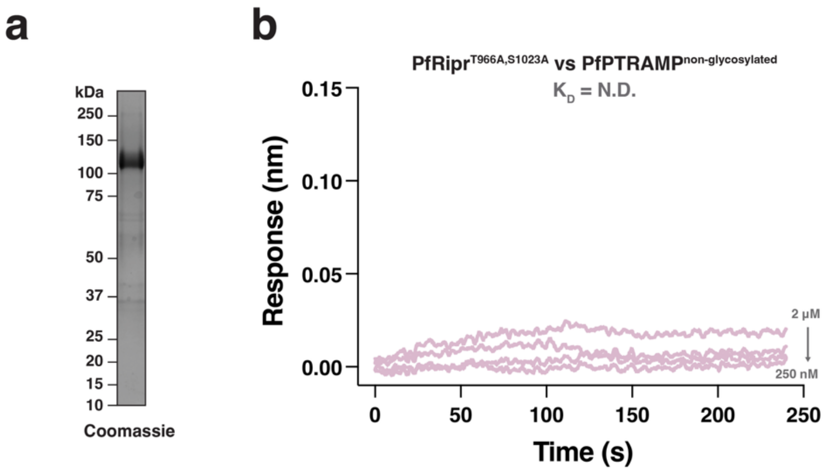

Supplementary Figure 2. Non-glycosylated forms of monomeric PfPTRAMP and PfRipr do not interact with one another. a. SDS-PAGE of purified recombinant $PfRipr^{T966A,S1023A.}$ Purification was repeated at least three times with similar results **b.** Biolayer interferometry sensorgram of $PfRipr^{T966A,S1023A}$ vs $PfPTRAMP^{non\text{-}glycosylated}$. Source data are provided as a Source Data file.

a

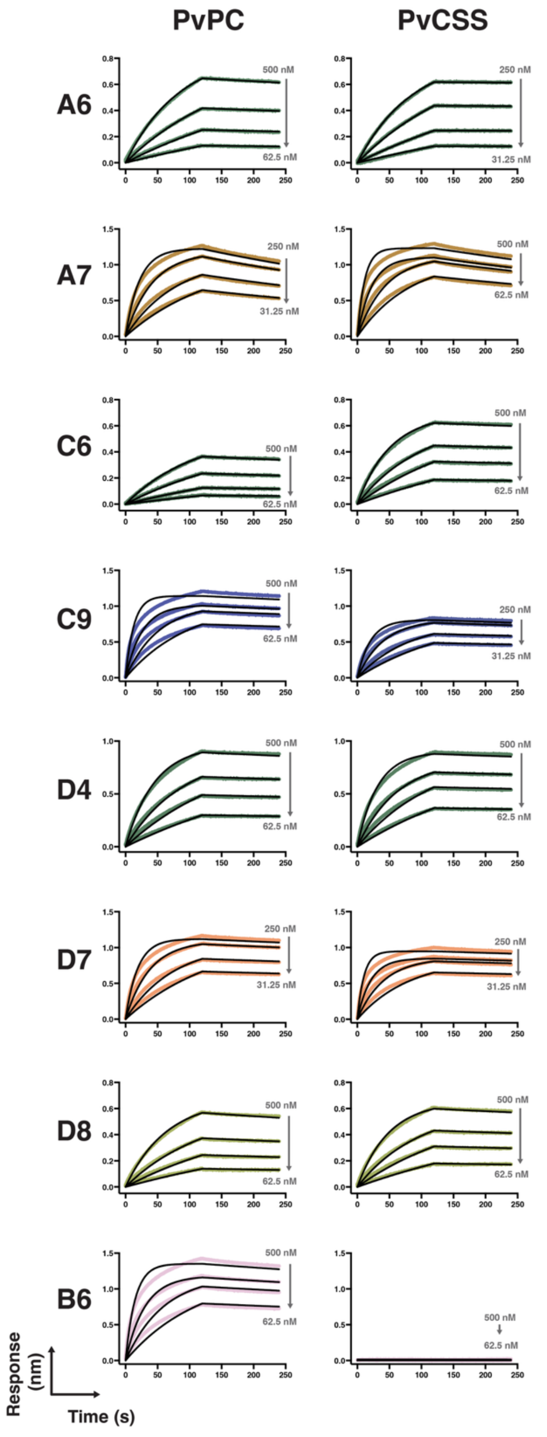

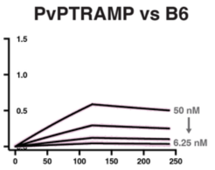

b

K_D (nM)

	PvPC	PvCSS	PvPTRAMP
A6	19.0	1.4	NT
A7	8.6	6.4	NT
B6	3.7	N.D	65.3
D4	7.0	3.9	NT
C6	17.0	8.2	NT
C9	2.4	1.9	NT
D7	1.8	1.5	NT
D8	19.2	9.8	NT

c

Nanobody 2 (columns); Nanobody 1 (rows)

Nanobody 1	A6	C6	D4	A7	D7	D8	C9	B6	PvRipr
A6	11	27	84	4	46	6	32	30	45
C6	-76	-69	73	-84	37	-75	-52	-56	44
D4	27	29	-6	28	41	38	59	55	49
A7	16	37	100	7	50	10	55	39	-7
D7	41	62	94	38	-7	47	74	68	45
D8	11	29	98	3	54	7	44	33	54
C9	-19	-4	67	-14	43	-13	4	12	22
B6	0	13	71	-6	41	-2	39	20	39

d

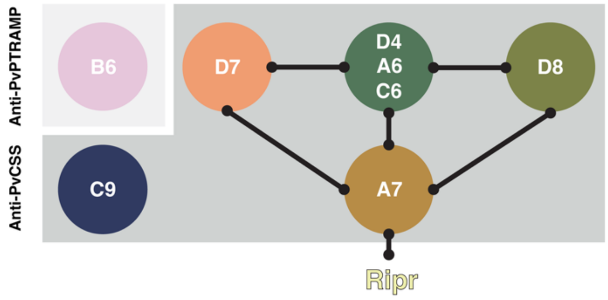

Supplementary Figure 3. Characterisation of anti-PvPC nanobodies. a. Biolayer interferometry sensorgrams of nanobodies binding to PvPC, PvCSS or PvPTRAMP. Data are shown in color and the 1:1 model best fit shown in black. **b.** Table of K_D values for nanobodies against PvPC and PvCSS. N.D = not determined, NT= not tested. **c-d.** Epitope binning of nanobodies. Boxes are colored on a sliding scale where red represents a competing nanobody and blue represents no competition. Epitope bins from competition with PvRipr are represented in (D). The competition table has been normalized to the greatest response, which was arbitrarily set to 100. Nanobodies that compete with one another or with Ripr binding are connected with black bars. Source data are provided as a Source Data file.

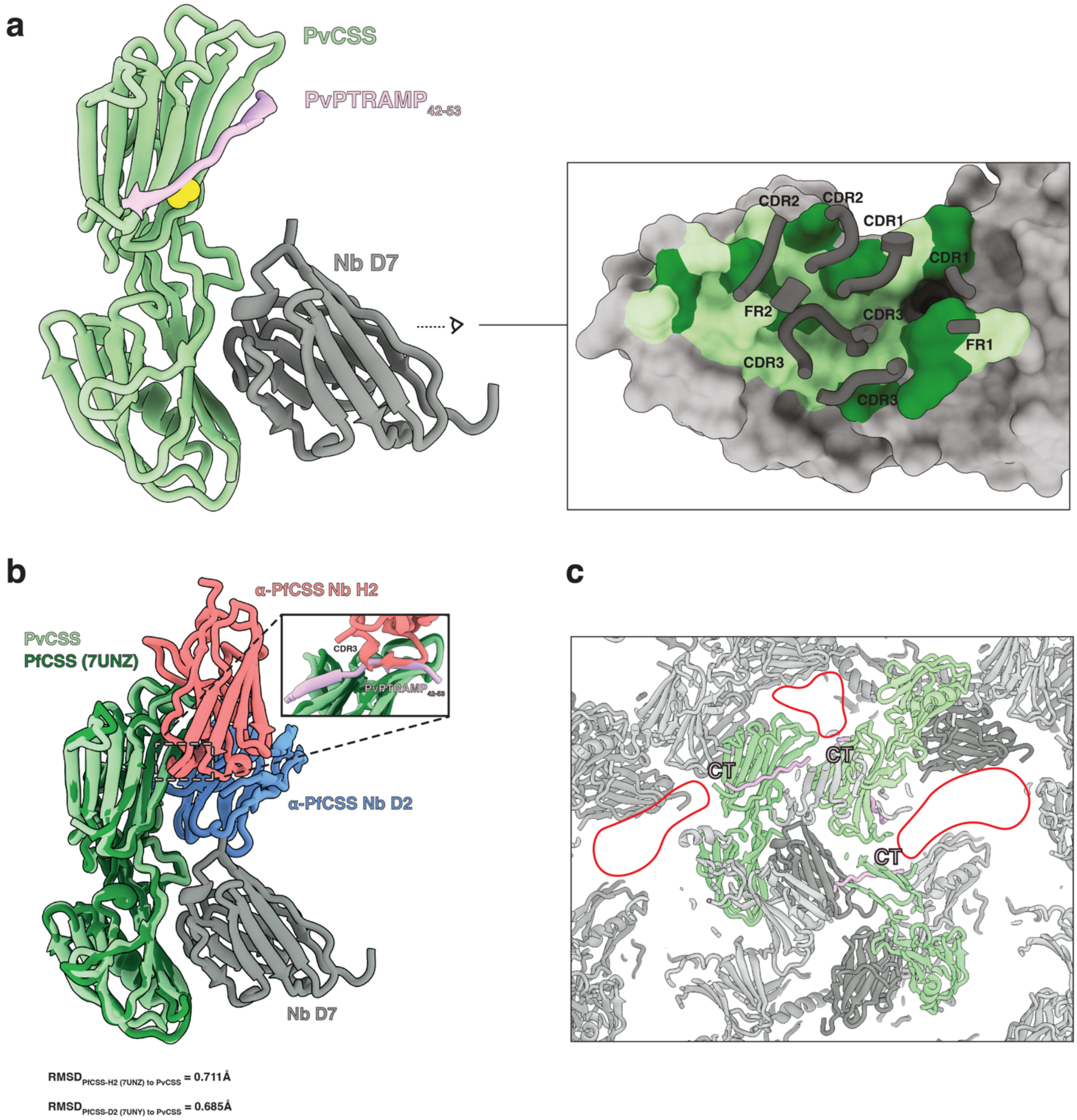
a
PvCSS
PvPTRAMP42-53
Nb D7
CDR2
CDR2
CDR1
CDR1
FR2
CDR3
FR1
CDR3
CDR3
b
α-PfCSS Nb H2
PvCSS
PfCSS (7UNZ)
CDR3
PvPTRAMP42-53
α-PfCSS Nb D2
Nb D7
RMSD PfCSS-H2 (7UNZ) to PvCSS = 0.711Å
RMSD PfCSS-D2 (7UNY) to PvCSS = 0.685Å
c
CT
CT
CT

Supplementary Figure 4. Comparison of PvCSS crystal structure with PfCSS structures. a. Binding interface between nanobody D7 and PvCSS. The detail shows the entire interface. PvCSS is grey and the region of D7 binding is shown in pale green, with hydrogen bonds and salt bridges shown in forest green. Regions of D7 involved in the interaction are shown and consist of complementarity determining regions (CDR) and framework regions (FR). Regions of D7 not involved in binding are omitted for clarity. **b.** Overlay of PfCSS-H2 (H2 in red) and PfCSS-D2 (D2 in blue) crystal structures with PvCSS-PvPTRAMP-D7(D7 in grey) crystal structure. Inset shows $PvPTRAMP_{42-53}$ occupies the same region as the CDR3 loop of H2, explaining how it competes with PfPTRAMP binding to PfCSS[1]. **c.** Crystal packing of PvCSS-PvPTRAMP-D7. The asymmetric unit is shown in color (PvCSS in green, PvPTRAMP in pink and D7 in dark grey) and other symmetry related copies in light grey. Red circles show pockets within the crystal lattice that are adjacent to the C-terminal (CT) of $PvPTRAMP_{42-53}$ that likely accommodate the GFD and TSR domains of PvPTRAMP.

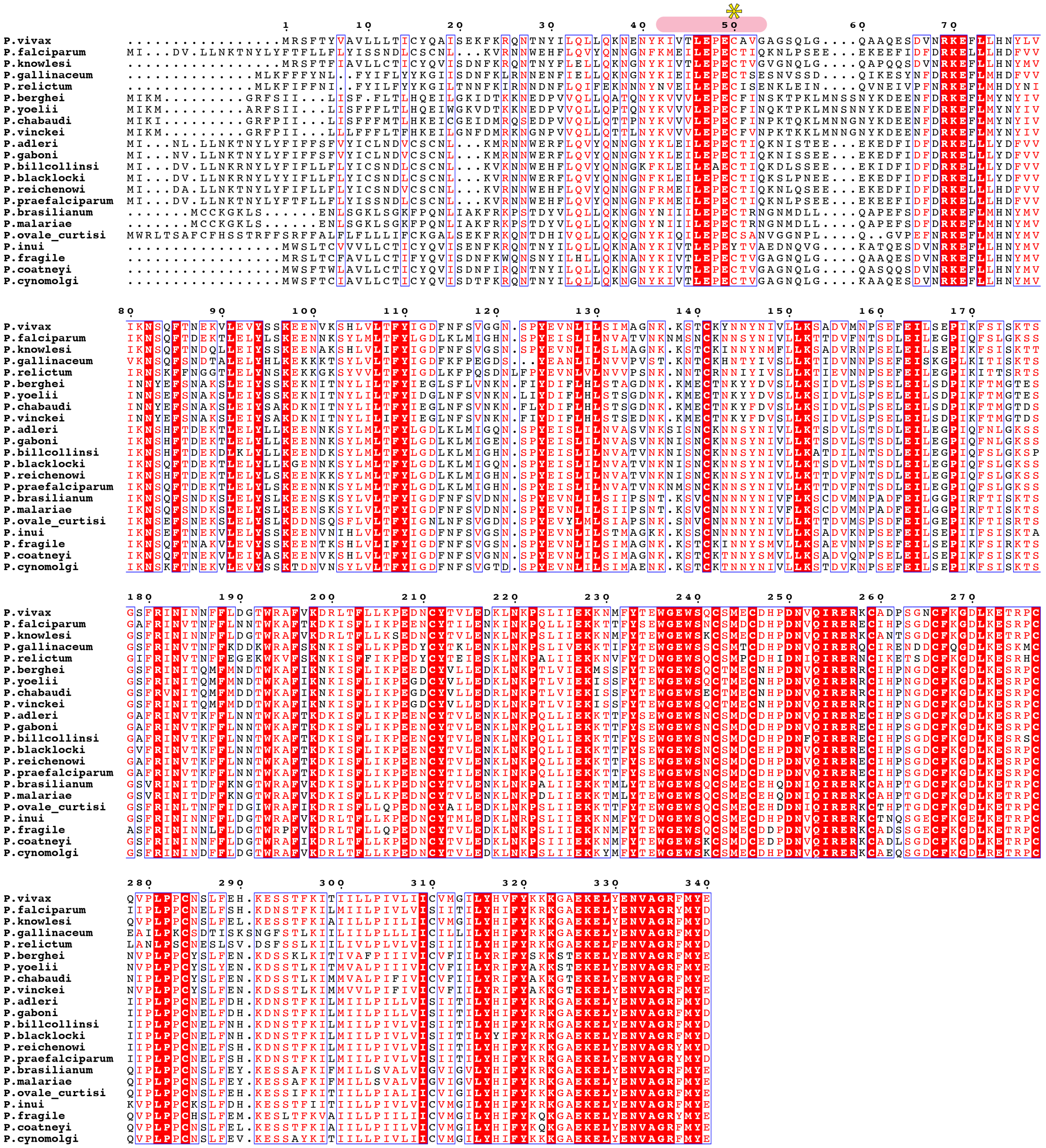

Supplementary Figure 5. Comparison of PTRAMP sequences from a selection of *Plasmodium* species. Multiple sequence alignment of PTRAMP from several *Plasmodium* species. Regions involved in PvCSS binding are indicated with pink above the interacting amino acids. The unpaired cysteine involved in intermolecular disulfide formation is marked with a yellow asterisk.

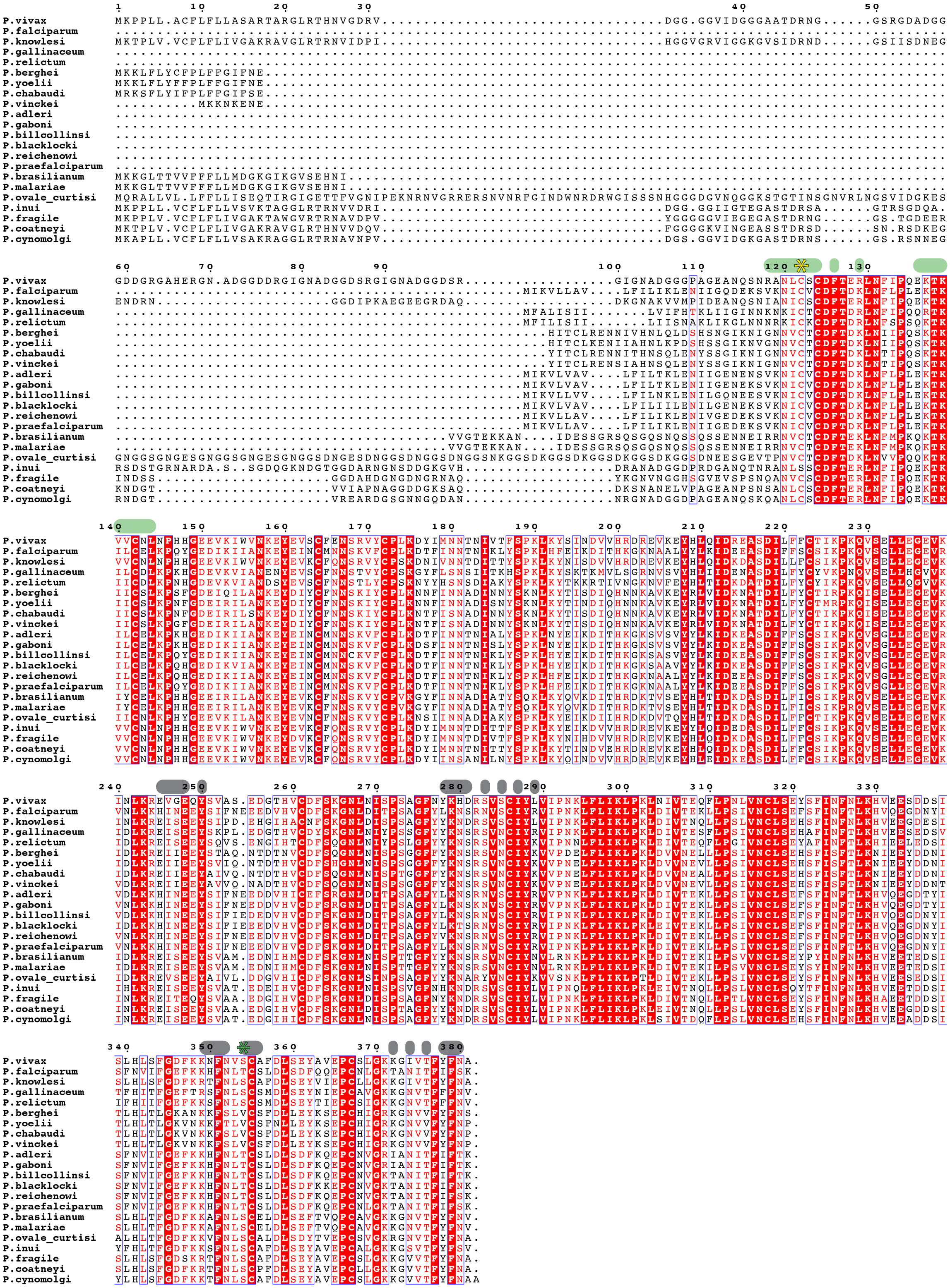
P.vivax
P.falciparum
P.knowlesi
P.gallinaceum
P.relictum
P.berghei
P.yoelii
P.chabaudi
P.vinckei
P.adleri
P.gaboni
P.billcollinsi
P.blacklocki
P.reichenowi
P.praefalciparum
P.brasilianum
P.malariae
P.ovale_curtisi
P.inui
P.fragile
P.coatneyi
P.cynomolgi

Supplementary Figure 6. Comparison of CSS sequences from a selection of Plasmodium species. Multiple sequence alignment of CSS from several Plasmodium species. Regions involved in PvPTRAMP binding are indicated by green above the interacting amino acids. Regions involved in the D7 interface are marked grey. The unpaired cysteine involved in intermolecular disulfide formation is marked with a yellow asterisk. The Ser354 that was mutated to remove a potential N-linked glycan is marked by a green asterisk.

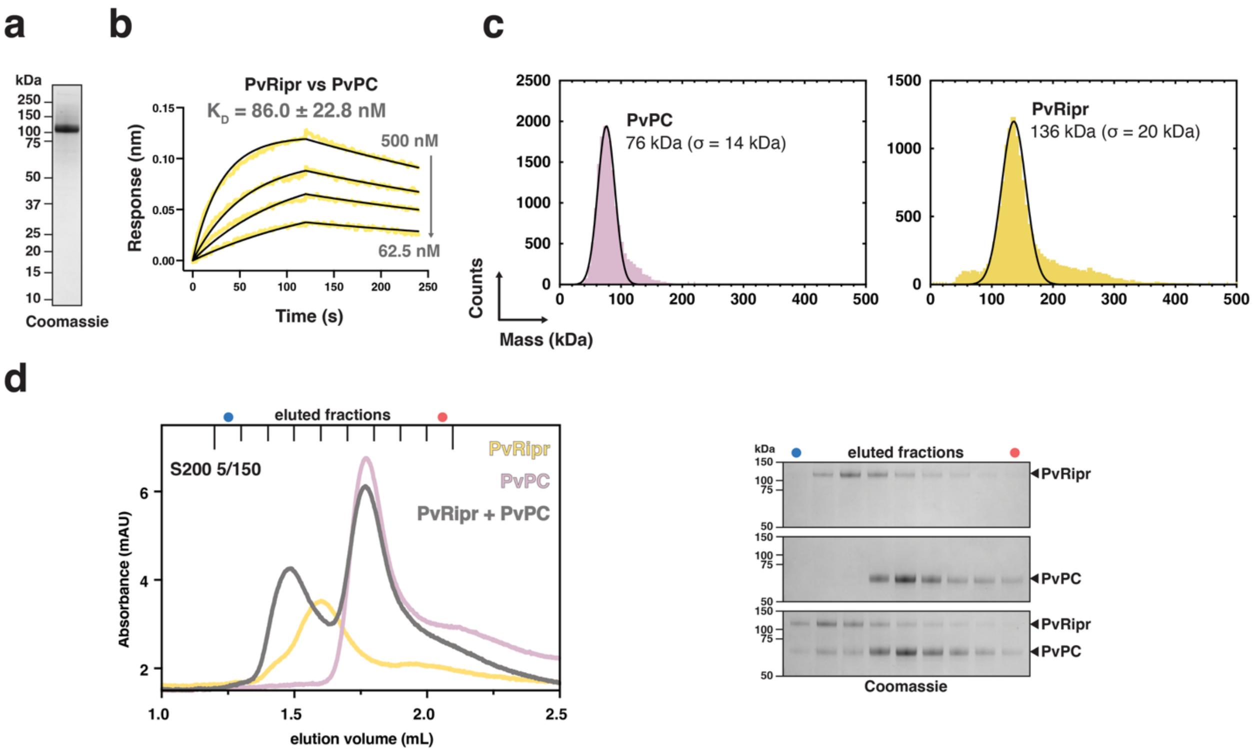

Supplementary Figure 7. The PvPCR complex can be reconstituted *in vitro* using recombinant proteins. a. Representative SDS-PAGE of recombinant full-length PvRipr. Purification was repeated at least three times with similar results **b.** Representative biolayer interferometry sensorgram of PvRipr vs PvPC. Data are in yellow and 1:1 model best fit in black. **c.** Mass distribution plots of PvPC (pink) and PvRipr (yellow) as determined by mass photometry. Histogram data are in color and the Gaussian curve fit in black. **d.** Size-exclusion chromatography of PvPC (pink), PvRipr (yellow) and PvPCR (grey), with SDS-PAGE results of the co-complexation, showing co-elution of PvPC and PvRipr. Co-complexation was repeated twice with similar results. Source data are provided as a Source Data file.

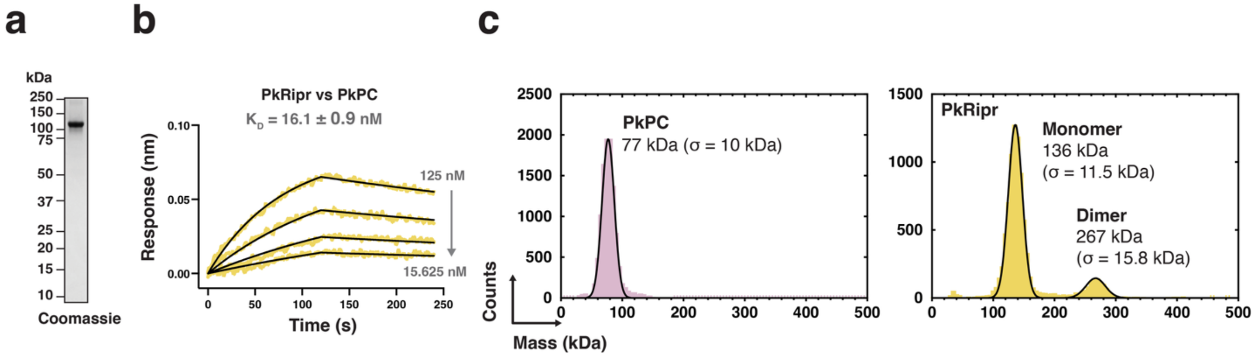

Supplementary Figure 8. Biophysical analysis of PkPC and PkRipr. a. SDS-PAGE of recombinant full-length PkRipr. Purification was repeated at least twice with similar results **b.** Representative biolayer interferometry sensorgram of PkRipr vs PkPC. Data are in yellow and 1:1 model best fit in black. **c.** Mass distribution plots of PkPC (pink) and PkRipr (yellow) as determined by mass photometry. Histogram data are in color and the Gaussian curve fit in black**.** Source data are provided as a Source Data file.

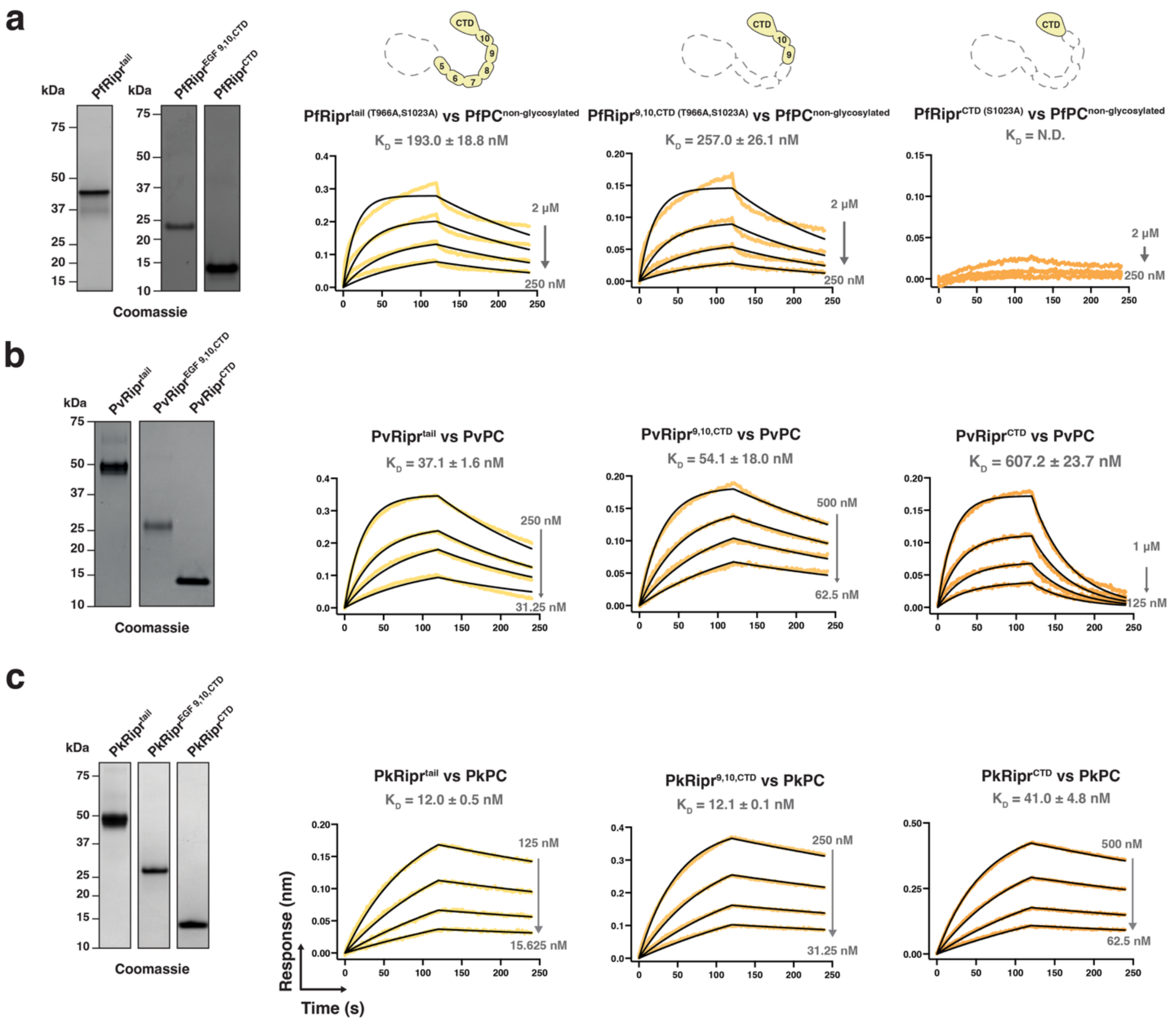

Supplementary Figure 9. Biophysical analysis of Ripr truncations. a. SDS-PAGE of recombinant PfRipr truncations and representative biolayer interferometry sensorgrams of Ripr truncations vs PfPC. $PfRipr^{tail}$ and $PfRipr^{EGF\ 9,10,CTD}$ harbor T966A and S1023A mutations and $PfRipr^{CTD}$ harbors S1023A mutation. **b.** SDS-PAGE of recombinant PvRipr truncations and representative biolayer interferometry sensorgrams of Ripr truncations vs PvPC. **c.** SDS-PAGE of recombinant PkRipr truncations and representative biolayer interferometry sensorgrams of Ripr truncations vs PkPC. For all sensorgrams, data are in color and 1:1 model best fit in black where applicable. Schematics of the Ripr truncations are shown above. All purifications were repeated at least twice with similar results. Source data are provided as a Source Data file.

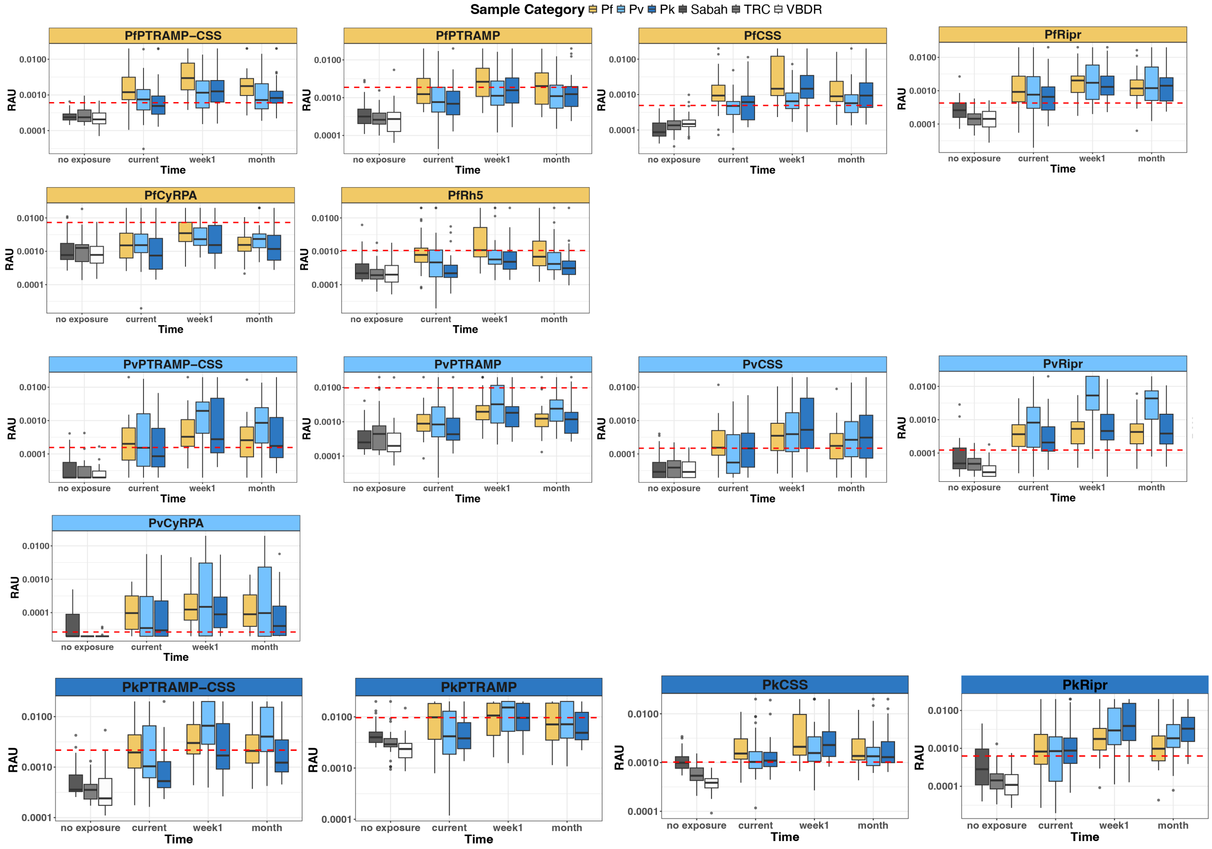

Supplementary Figure 10. Serological assessment of invasion antigens. Antibody kinetics in longitudinal plasma samples collected from individuals with *P. falciparum* (yellow), *P. vivax* (light blue), *P. knowlesi* (dark blue) infections at three timepoints (current (n=95), week 1 (n=98) and a month (n=98)) on a logarithmic scale. Samples from Volunteer Biospecimen Donor Registry (white, n =28) and Thailand Red Cross (light grey, n = 29) was used to set the sero-positivity cut-off (red dotted line), which is mean of these samples + 2 x standard deviation. Samples from healthy (non-febrile) individuals from Sabah Malaysia (dark grey, n=30) are not included in the sero-positivity calculation as their previous exposure status to any of the *Plasmodium* species is unknown. Boxplots display the median (horizontal line), interquartile range (IQR) (the box), largest and smallest values (1.25 x IQR, whiskers) and outliers are displayed as points. Source data are provided as a Source Data file.

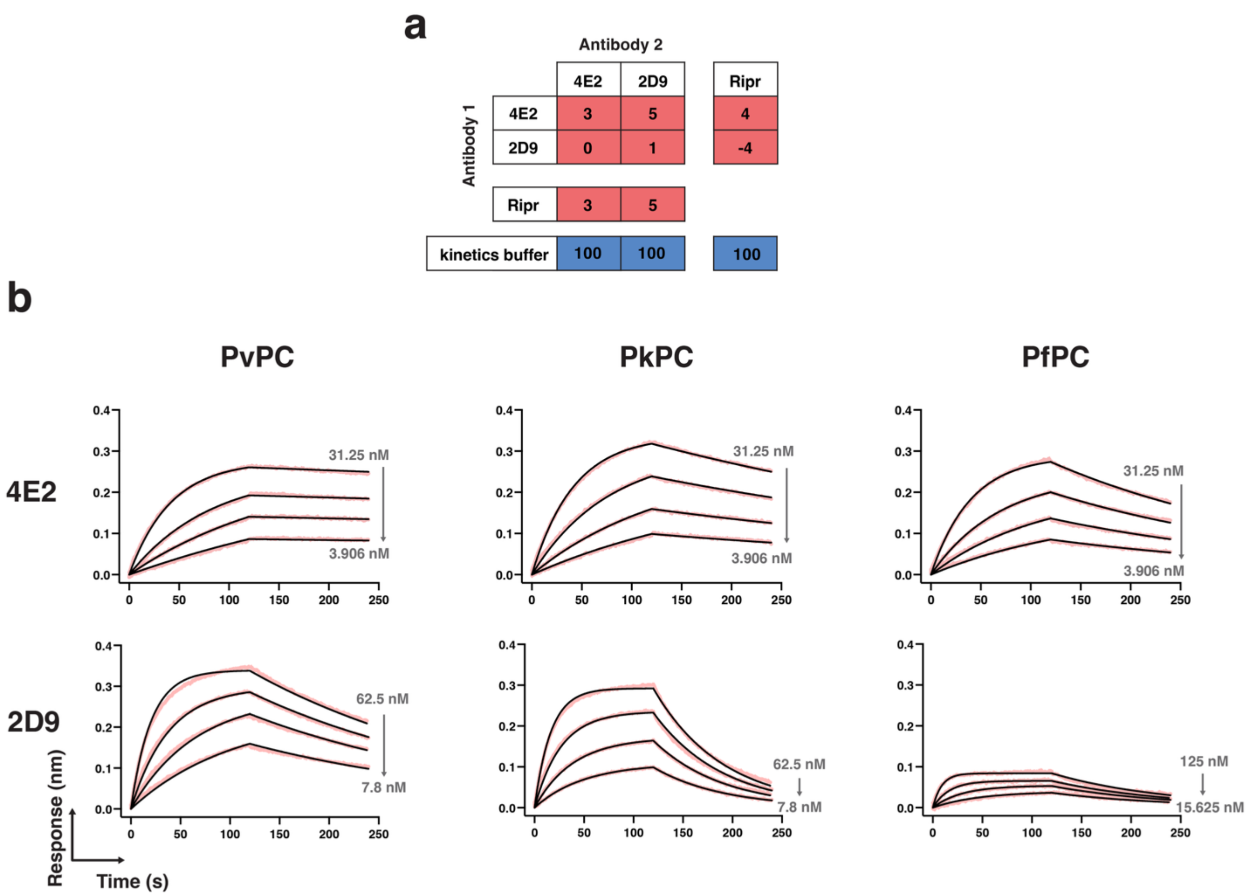

c

K_D (nM)

	PvPC	PkPC	PfPC
**4E2**	0.4	2.6	3.6
**2D9**	6.3	22.1	11.8

Supplementary Figure 11. Characterization of anti-PvPC mouse monoclonal antibodies. a. Competition binning of anti-PvPC monoclonal antibodies and PvRipr. **b.** Representative biolayer interferometry sensorgrams of antibody binding to PvPC, PkPC, and PfPC. Data are in color and 1:1 model best fit in black. **c.** Table of K_D values, in nM, for the curves in b. Source data are provided as a Source Data file.

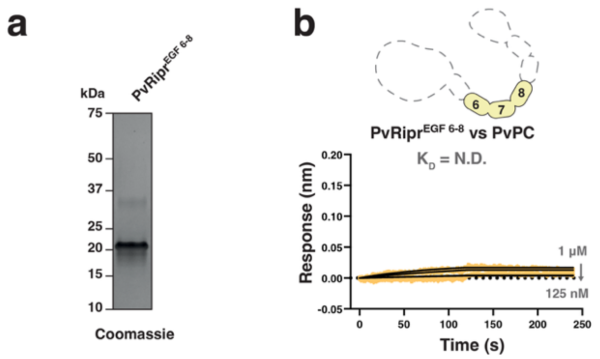

c

Antibody 2 (rows: Antibody 1)

Antibody 1	5B3	5B4	4H10	6A10	5E11	PvPC
5B3	3	93	59	93	62	33
5B4	98	3	58	85	56	33
4H10	94	92	3	84	55	1
6A10	100	91	55	2	56	33
5E11	98	95	61	87	1	33

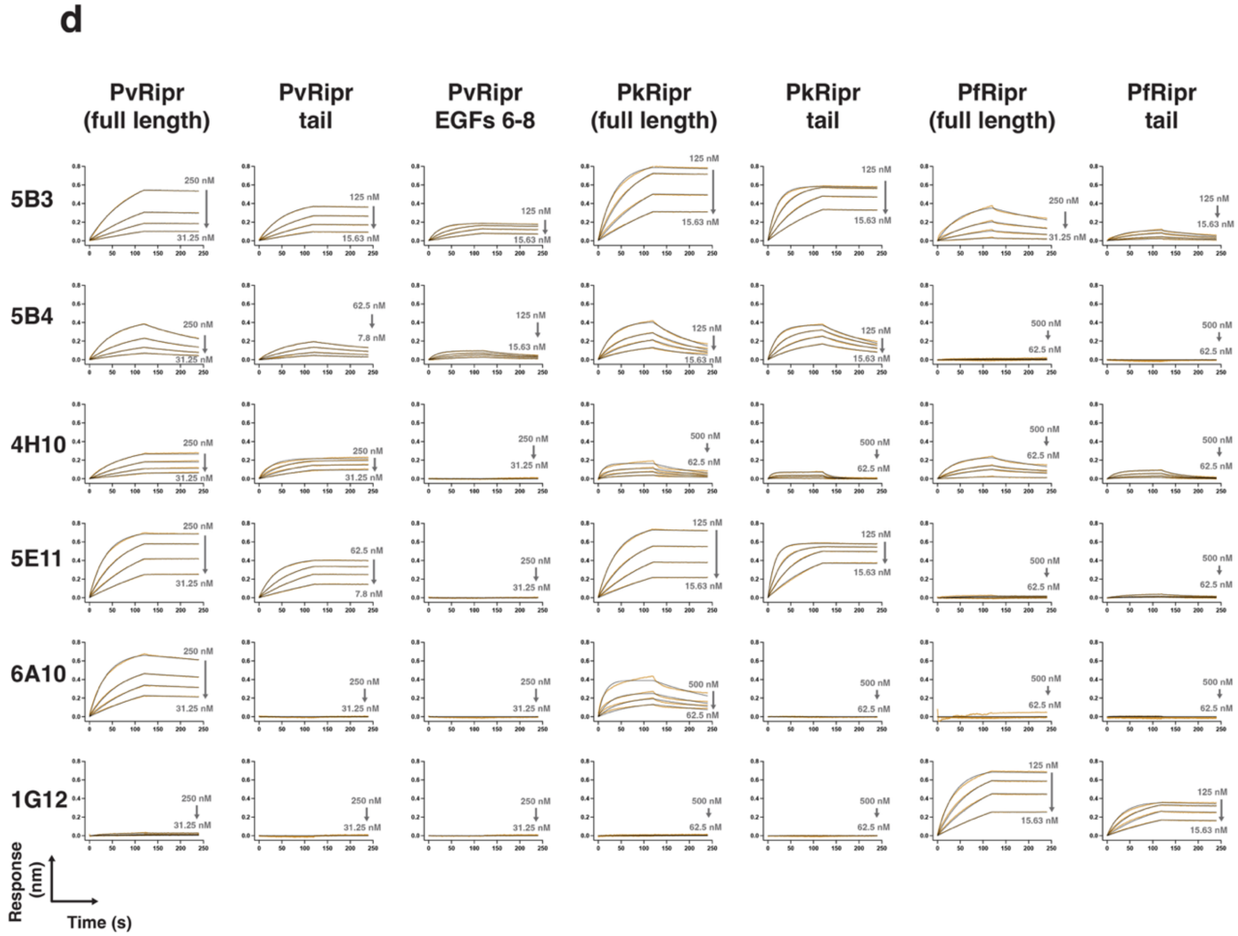

e

	PvRipr full length	PvRipr tail	PvRipr EGF 6-8	PkRipr full length	PkRipr tail	PfRipr full length	PfRipr tail
5B3	2.7	1.1	1.3	20.4	0.4	53.3	40.9
5B4	71.2	15.5	14.8	30.0	17.4	N.D	N.D
4H10	low nM	low nM	N.D	52.7	241.7	151.1	270.7
5E11	low nM	low nM	N.D	0.5	0.13	N.D	N.D
6A10	3.5	N.D	N.D	44.9	N.D	N.D	N.D
1G12	N.D	N.D	N.D	N.D	N.D	0.3	1.4

Supplementary Figure 12. Characterization of anti-PvRipr mouse monoclonal antibodies. a. SDS-PAGE of recombinant $PvRipr^{EGF6-8}$. Purification was repeated at least three times with similar results **b.** Biolayer interferometry sensorgram of $PvRipr^{EGF6-8}$ vs PvPC showing no interaction between the two proteins. **c.** Competition binning of anti-PvRipr antibodies. Only 4H10 interferes with PvPCR complex formation. **d.** Representative biolayer interferometry sensorgrams of antibody binding to PvRipr, PkRipr and PfRipr. The anti-PfRipr antibody 1G12 was included as a control[2]. Data are in color and 1:1 model best fit in black. **e.** Table of K_D values, in nM, for the curves in d. N.D. = not determined. Source data are provided as a Source Data file.

a

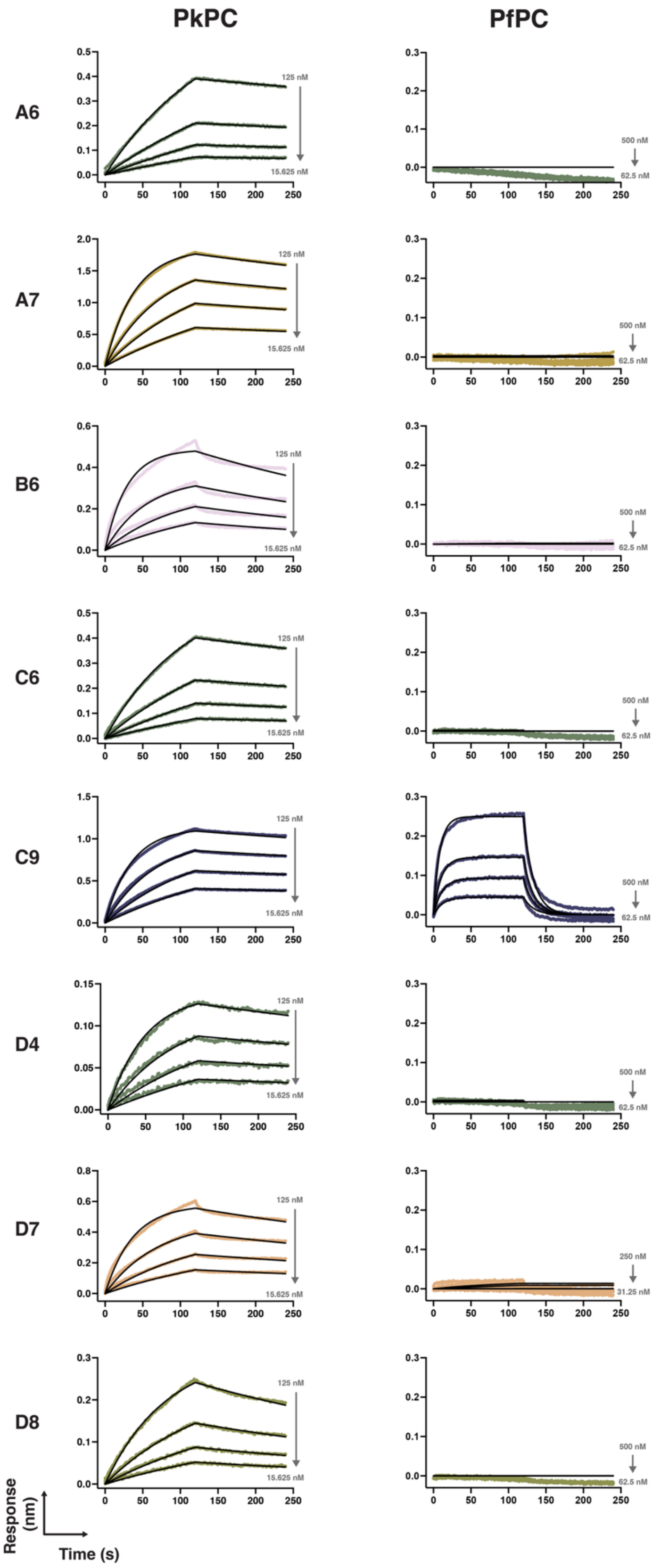

b

K_D (nM)

	PkPC	PfPC
**A6**	14	N.D
**A7**	4.4	N.D
**B6**	9.0	N.D
**C6**	13.8	N.D
**C9**	2.7	822
**D4**	6.1	N.D
**D7**	7.0	N.D
**D8**	25.4	N.D

Supplementary Figure 13. Anti-PvPC nanobodies broadly cross-react with PkPC, but not PfPC. a. Representative biolayer interferometry sensorgrams of nanobody binding to PkPC and PfPC. Data are in color and 1:1 model best fit in black. **b.** Table of K_D values, in nM, for the curves in a). N.D. = not determined. Source data are provided as a Source Data file.

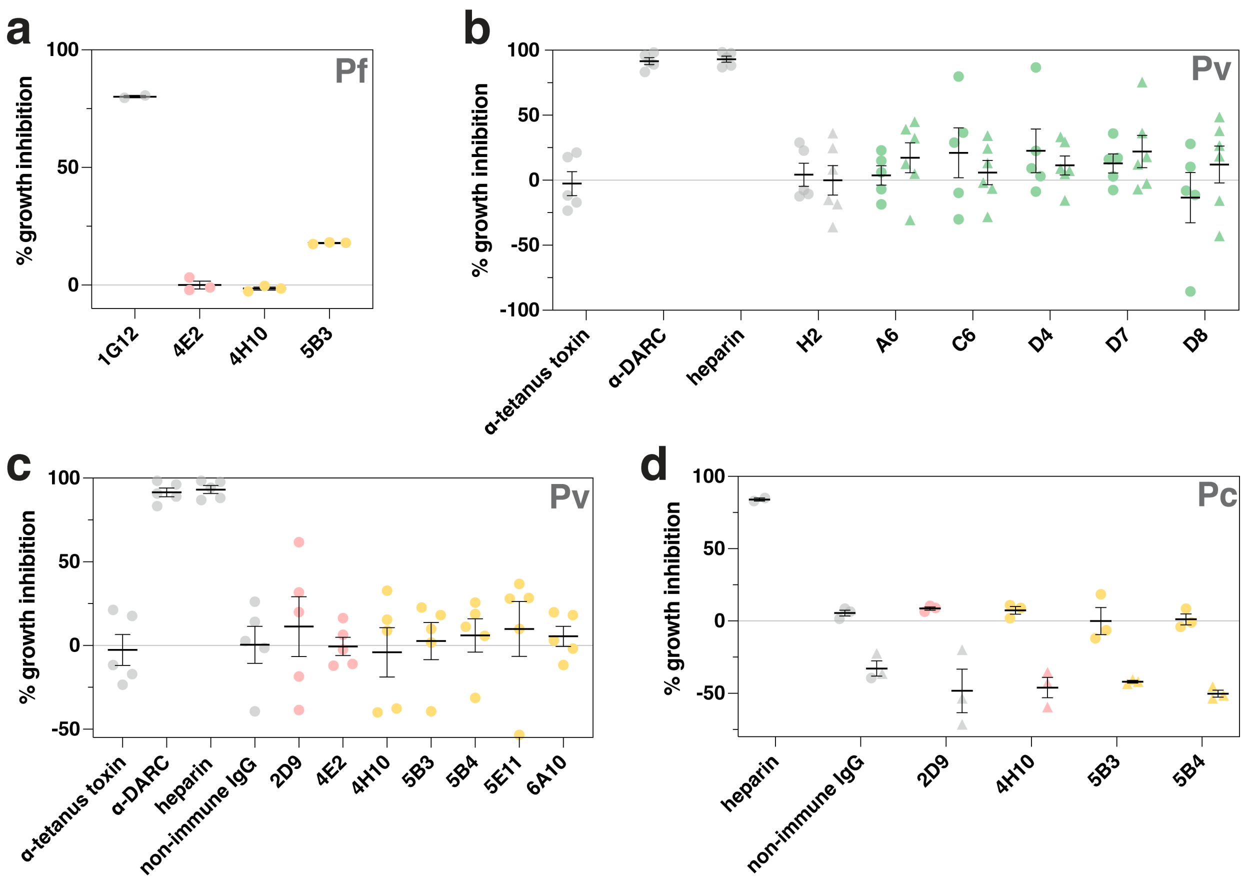

Supplementary Figure 14. Growth-inhibition assays of antibodies and nanobodies against *P. falciparum*, *P. vivax*, and *P. cynomolgi* a. Initial screening of cross-reactive antibodies against *P. falciparum.* Antibodies were tested at a final concentration of 0.5 mg/mL for 4E2, 4H10, and 5B3, and at 1 mg/mL for 1G12 (anti-PfRipr antibody). Three biological replicates are plotted for anti-PvPC and anti-PvRipr antibodies, and two biological replicates are plotted for 1G12 **b.** *Ex vivo* growth inhibition assay of *P. vivax* parasites testing anti-PvCSS nanobodies. Circles and triangles represent 100 µg/mL and 0.5 mg/mL final concentration, respectively. Data are from five (100 µg/mL) and six (0.5 mg/mL) independent experiments. The anti-PfCSS nanobody, H2, was used as a negative control **c.** *Ex vivo* growth inhibition assay of *P. vivax* parasites. Antibodies were tested at a final concentration of 100 µg/mL. Data are from five independent experiments **d.** *P. cynomolgi* growth inhibition assay of anti-PvPC and anti-PvRipr antibodies. Three independent experiments were performed. Circles and triangles represent 125 µg/mL and 2 mg/mL final concentration, respectively. All panels show the mean and SEM. Source data are provided as a Source Data file.

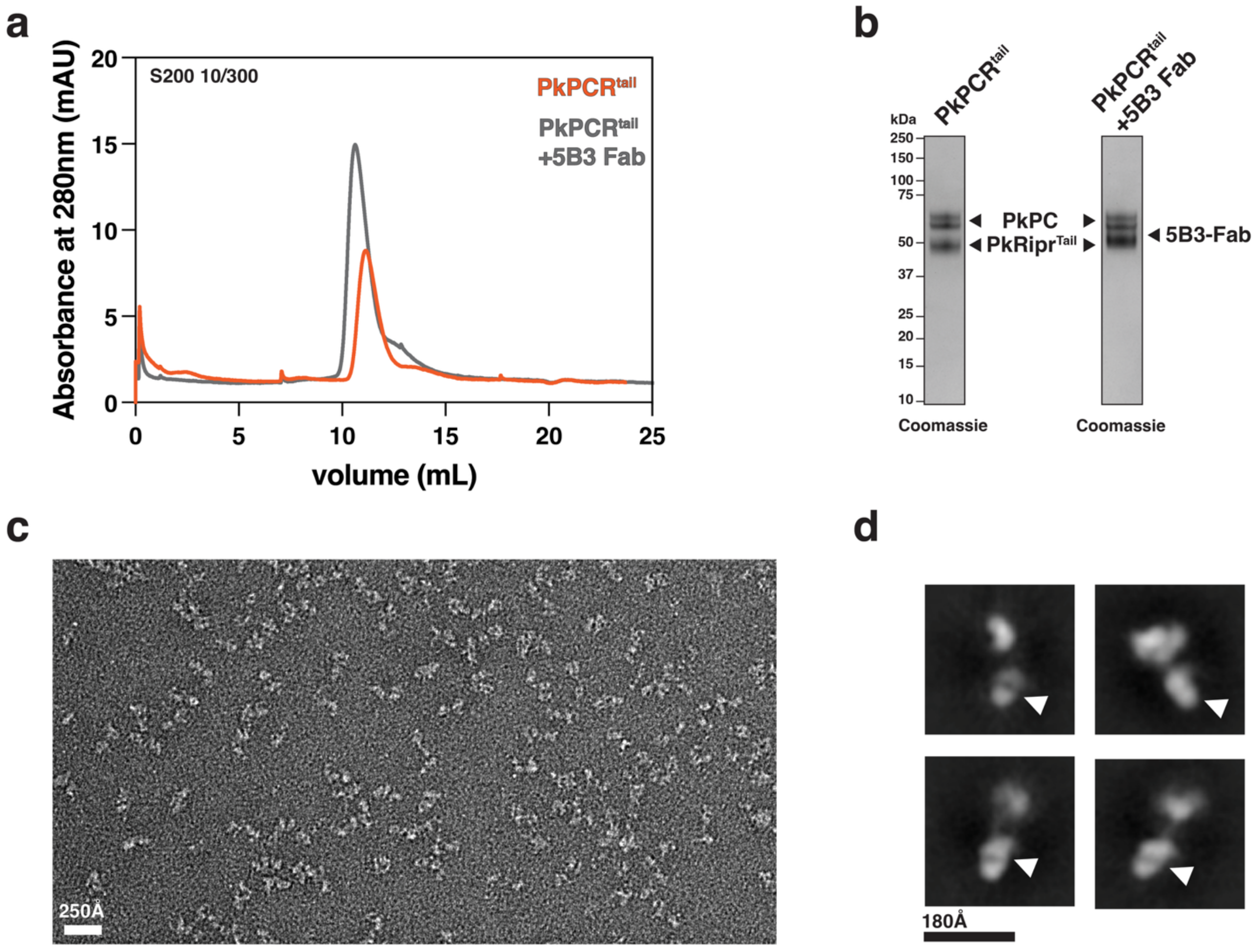

Supplementary Figure 15. Negative stain electron microscopy of PkPCR^{tail}+5B3 Fab shows an elongated structure. a. Size-exclusion chromatography chromatograph of PkPCR^{tail} and PkPCR^{tail}+5B3 Fab. **b.** Non-reducing SDS-PAGE of peak fractions of purified complexes from a. 5B3 Fab and Ripr^{tail} have approximately the same molecular weight. Purification of both complexes was carried out at least three times with similar results. **c.** Representative electron micrograph of negatively stained PkPCR^{tail}+5B3 Fab particles. **d.** Two-dimensional class averages of PkPCR^{tail}+5B3 Fab. White arrows show density attributable to 5B3-Fab. Source data are provided as a Source Data file.

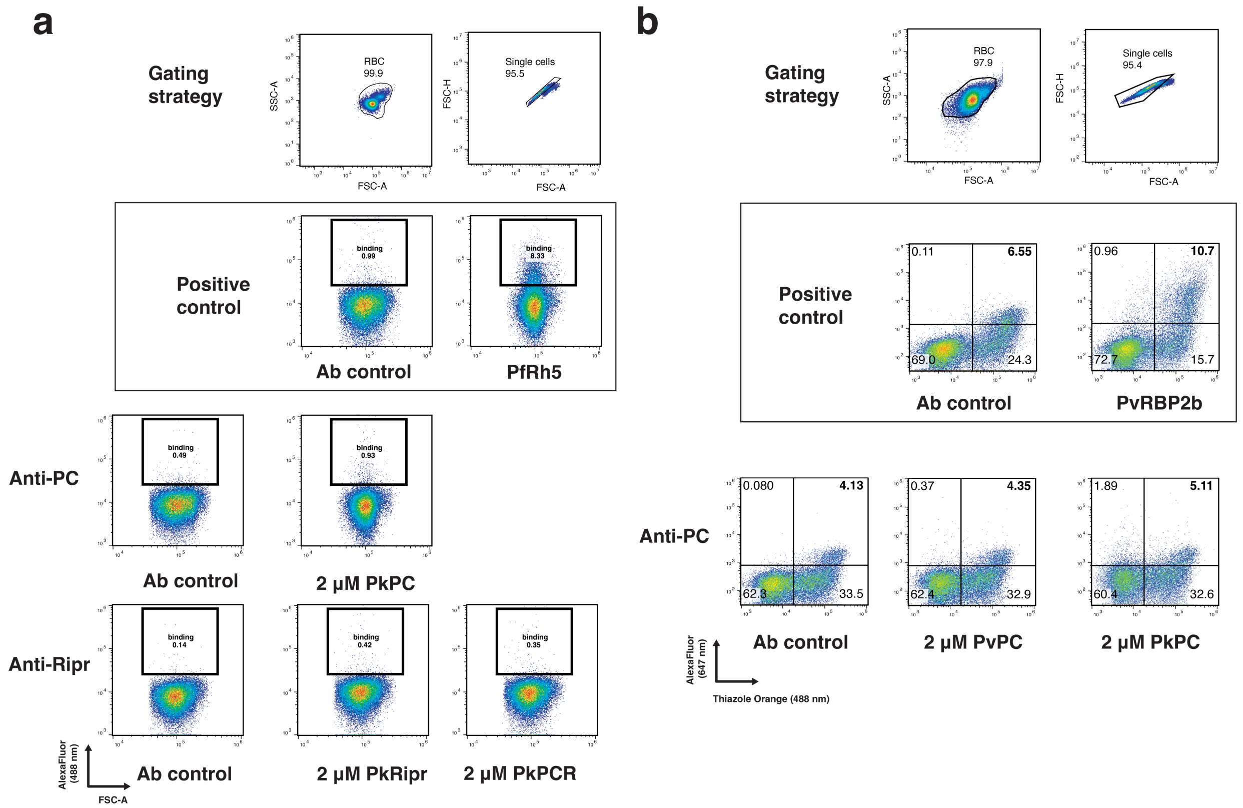

Supplementary Figure 16. PvPC, PkPC and PkPCR do not bind erythrocytes. a. Gating strategy and representative flow cytometry plots of PkPC, PkRipr, and PkPCR incubated with erythrocytes and detected with either anti-PC or anti-Ripr antibodies. PfRh5 was used as a positive control for erythrocyte binding. **b.** Gating strategy and representative flow cytometry plots of PvPC and PkPC incubated with reticulocyte enriched cord blood and detected with anti-PC antibody. PvRBP2b was used as a positive control for reticulocyte binding. Source data are provided as a Source Data file.

Supplementary table 1. Data collection and refinement statistics of PvCSS-PvPTRAMP-D7 structure

	**PvCSS_PvPTRAMP_D7**
**Beamline**	MX2
**Wavelength**	0.953739
**Resolution range**^	48.32 - 3.14 (3.252 - 3.14)
**Space group**	P 32
**Cell dimensions**	
**a,b,c (Å)**	125.2, 125.2, 106.5
**α, β, γ (°)**	90, 90, 120
**Total reflections**	174, 965 (17,387)
**Unique reflections**	32, 570 (3,251)
**Multiplicity**	5.4 (5.4)
**Completeness (%)**	99.9 (99.9)
**<I/σ I>**	13.2 (1.5)
**R_{merge} (%)**	9.6 (104.5)
**R_{pim} (%)**	4.6 (49.7)
**$CC_{1/2}$ (%)**	99.8 (54.4)
**Reflections used in refinement**	32, 565 (3, 250)
**Reflections used for R-free**	1, 700 (215)
**R_{work} (%)**	22.5 (35.4)
**R_{free} (%)**&	27.3 (37.3)
**Number of non-hydrogen atoms**	9, 551
**macromolecules**	9, 551
**Protein residues**	1, 196
**RMS (bonds) (Å)**	0.003
**RMS (angles) (°)**	0.67
**Ramachandran plot**	
**Ramachandran favored (%)**	92.2
**Ramachandran allowed (%)**	7.8
**Ramachandran outliers (%)**	0.0
**Rotamer outliers (%)**	1.03
**Clashscore**	7.3
**Wilson B-factor**	97.5
**Average B-factor**	93.6

^ Statistics for the highest-resolution shell are shown in parentheses.
& 5% of data were used for R_{free} calculation

Supplementary table 2. Table of contacts between PvCSS and PvPTRAMP

PvCSS Residue (BSA Å²)	Interaction type	PvPTRAMP residue
**Arg118 (23.8)**		
Arg	VDW	Gly53
Arg^{N}	HB	$Gly53^{O}$
**Ala119 (21.2)**		
Ala	VDW	Val52, Gly53
Ala^{N}	HB	$Gly53^{O}$
Ala^{O}	HB	$Gy53^{N}$
**Asn120 (26.0)**		
Asn	VDW	Ala51, Val52, Gly53
**Leu121 (52.9)**		
Leu	VDW	Cys50, Ala51, Val52, Gly53
Leu^{N}	HB	$Ala51^{O}$
Leu^{O}	HB	$Ala51^{N}$
**Cys122 (38.0)**		
Cys	VDW	Glu49, Cys50, Ala51
$Cys^{S\gamma}$	DSB	$Cys50^{S\gamma}$
**Ser123 (41.1)**		
Ser	VDW	Pro48, Glu49, Cys50
Ser^{N}	HB	$Glu49^{O}$
**Cys124 (3.7)**		
Cys	VDW	Glu47, Glu49
**Phe126 (6.3)**		
Phe	VDW	Glu47
**Arg129 (42.2)**		
Arg	VDW	Leu46, Glu47
**Glu136 (26.9)**		
Glu	VDW	Ile43
**Lys137 (62.1)**		
Lys	VDW	Lys42, Ile43
Lys^{O}	HB	$Ile43^{N}$
**Thr138 (27.9)**		
Thr	VDW	Ile43
**Lys139 (77.0)**		
Lys	VDW	Lys42, Ile43, Val44, Thr45
Lys^{N}	HB	$Ile43^{O}$
Lys^{O}	HB	$Thr45^{N}$
**Val140 (35.8)**		
Val	VDW	Ile43, Thr45, Glu47

**Val141 (57.8)**		
Val	VDW	Val44, Thr45, Leu46, Glu47
Val^{N}	HB	$Thr45^{O}$
Val^{O}	HB	$Glu47^{N}$
**Cys142 (34.2)**		
Cys	VDW	Glu47, Pro48
**Asn143 (21.5)**		
Asn	VDW	Pro48
**Leu144 (4.0)**		
Leu	VDW	Glu49

Supplementary table 3. Table of contacts between PvCSS and nanobody D7

PvCSS Residue (BSA Å^2)	Interaction type	Nb_D7 residue^
**Glu245 (41.9)**		
Glu	VDW	Arg31
Glu^{O}	HB	Arg31$^{N\varepsilon}$
**Val246 (17.1)**		
Val	VDW	Arg31
**Gly247 (33.2)**		
Gly	VDW	Arg31, Tyr32
**Glu248 (94.0)**		
Glu	VDW	Arg31, Ala33, Asn52, Ser52A, Phe98
Glu^{N}	HB	Arg31^{O}
Glu$^{O\varepsilon2}$	HB	Ser52A^{N}, Ser52A$^{O\gamma}$
**Tyr250 (64.1)**		
Tyr	VDW	Asn52, Asp53, Phe56
Tyr^{OH}	HB	Asn52$^{N\delta2}$, Asp53$^{O\delta2}$
**Tyr278 (3.5)**		
Tyr	VDW	Tyr102
**Lys279 (66.00)**		
Lys	VDW	Lys94, Gln96, Tyr100, Asp101, Tyr102
Lys$^{N\zeta}$	SB	Asp101$^{O\delta1}$
**His280 (121.3)**		
His	VDW	Val2, Phe27, Tyr32, Lys94, Asp101, Tyr102
His^{N}	HB	Tyr102^{OH}
His^{O}	HB	Lys94$^{N\zeta}$
His$^{N\delta1}$	HB	Tyr32^{OH}
**Asp281 (10.5)**		
Asp	VDW	Tyr32, Lys94
Asp$^{O\delta1}$	HB	Tyr32^{OH}
**Ser283 (37.9)**		
Ser	VDW	Lys94, Gln96, Asp101
**Ser285 (19.7)**		
Ser	VDW	Gln96, Ala97, Phe98
**Ile287 (21.6)**		
Ile	VDW	Phe98
**Leu289 (23.2)**		
Leu	VDW	Phe56
**Lys349 (4.6)**		

Lys	VDW	Tyr58
**Asn350 (13.5)**		
Asn	VDW	Tyr58
Asn^{O}	HB	Tyr58^{OH}
**Phe351 (8.8)**		
Phe	VDW	Tyr58
Phe^{N}	HB	Tyr58^{OH}
**Asn352 (83.2)**		
Asn	VDW	Asp50. Tyr58, Phe98, Gly99
Asn$^{N\delta2}$	HB	Asp50$^{O\delta2}$
**Ala354 (43.6)**		
Ala	VDW	Phe98, Gly99, Tyr100
**Cys355 (1.7)**		
Cys	VDW	Tyr100
**Ala356 (13.8)**		
Ala	VDW	Tyr100
**Lys372 (36.3)**		
Lys	VDW	Gln96, Tyr100
Lys$^{N\zeta}$	HB	Gln96$^{O\varepsilon1}$, Tyr100^{OH}
**Ile374 (41.6)**		
Ile	VDW	Gln96, Ala97, Tyr100
**Thr376 (16.1)**		
Thr	VDW	Ala97, Phe98
Thr$^{O\gamma1}$	HB	Phe98^{O}
**Tyr378 (57.2)**		
Tyr	VDW	Phe56, Thr57, Tyr58, Phe98
Tyr^{OH}	HB	Thr57^{O}
**Phe379 (0.6)**		
Phe	VDW	Tyr58
**Asn380 (13.4)**		
Asn	VDW	Thr57, Tyr58

^D7 Residues are labelled according to the Kabat numbering system

Supplementary table 4. Data collection and refinement statistics of PvRipr EGF7-8 – 5B3 structure

	**PvRipr EGF7-8 – 5B3**
**Beamline**	MX2
**Wavelength**	0.95365
**Resolution range**^	48.64 - 2.078 (2.13 – 2.08)
**Space group**	P 21 21 2
**Cell dimensions**	
**a,b,c (Å)**	80.27, 81.746, 92.149
**α, β, γ (°)**	90, 90, 90
**Total reflections**	230,633 (14,583)
**Unique reflections**	65,080 (4,385)
**Multiplicity**	6.3 (5.9)
**Completeness (%)**	99.3 (94.8)
**<I/σ I>**	7.3 (1.0)
**R_{merge} (%)**	15.1 (139.1)
**R_{pim} (%)**	6.5 (61.1)
**$CC_{½}$ (%)**	99.6 (60.6)
**Reflections used in refinement**	36,935 (2,462)
**Reflections used for R-free**	2,000 (136)
**R_{work} (%)**	21.1 (31.3)
**R_{free} (%)**&	23.9 (32.2)
**Number of non-hydrogen atoms**	4,204
**macromolecules**	3,953
**Protein residues**	514
**RMS (bonds) (Å)**	0.002
**RMS (angles) (°)**	0.56
**Ramachandran plot**	
**Ramachandran favored (%)**	96.26
**Ramachandran allowed (%)**	3.74
**Ramachandran outliers (%)**	0.00
**Rotamer outliers (%)**	2.21
**Clashscore**	4.35
**Wilson B-factor**	32.47
**Average B-factor**	55.04

^ Statistics for the highest-resolution shell are shown in parentheses.
& 5% of data were used for R_{free} calculation

Supplementary table 5. Table of contacts between 5B3 and PvRipr EGF7-8.

EGF7-8 Residue (BSA Å^2)	Interaction Type	5B3 Residue^
**Lys777 (21.2)**		
Lys	VDW	H-Arg73
**Val778 (11.4)**		
Val	VDW	H-Ser74
**Val779 (50.4)**		
Val	VDW	H-Ser28, H-Arg73, H-Ser74, H-Asn76
**Gln780 (100.3)**		
Gln	VDW	H-Ile23, H-Val24, H-Ser25, H-Ser74, H-Phe75, H-Asn76
Gln^{N}	HB	H-Ser74^{O}
Gln$^{O\varepsilon1}$	HB	H-Val24^{O}
Gln$^{N\varepsilon2}$	HB	H-Val24^{O}, H-Phe75^{O}
**Asn781 (0.2)**		
Asn	VDW	H-Ser74
**Thr784 (17.2)**		
Thr	VDW	H-Ser28
**Cys785 (1.7)**		
Cys	VDW	H-Asp31
**Glu786 (58.6)**		
Glu	VDW	H-Ser28, H-Lys30, H-Asp31, H-Arg73
Glu$^{O\varepsilon1}$	HB, SB	H-Arg73^{NH2}
Glu$^{O\varepsilon2}$	HB	H-Ser28$^{O\gamma}$
**Cys787 (18.9)**		
Cys	VDW	H-Lys30, H-Asp31
Cys^{N}	HB	H-Asp31$^{O\delta1}$, H-Asp31$^{O\delta2}$
Cys^{O}	HB	H-Lys30$^{N\zeta}$
**Lys788 (0.6)**		
Lys	VDW	H-Lys30, H-Glu53
Lys^{O}	HB	H-Lys30$^{N\zeta}$
**Glu789 (45.4)**		
Glu	VDW	H-Lys30, H-Glu53, H-Arg73
Glu$^{O\varepsilon1}$	HB, SB	H-Arg73^{NH2}
**Asn790 (27.9)**		
Asn	VDW	H-Lys30, H-Glu53
Asn^{N}	HB	H-Glu53$^{O\varepsilon2}$
**Leu791 (9.8)**		
Leu	VDW	H-Lys30, H-Asp31, H-Glu53
Leu^{O}	HB	H-Lys30$^{N\zeta}$
**Lys792 (127.3)**		

Lys	VDW	H-Ile29, H-Lys30, H-Asp31, H-Tyr32, H-Tyr33, H-Asp52, H-Glu53, H-Tyr96, H-Tyr97
Lys$^{N\zeta}$	HB, SB	H-Asp52$^{O\delta1}$
Lys$^{N\zeta}$	SB	H-Asp52$^{O\delta2}$
Lys$^{N\zeta}$	HB	H-Lys30^{O}, H-Tyr32^{O}
**Arg793 (75.8)**		
Arg	VDW	H-Asp31, H-Tyr32, H-Tyr96, H-Tyr97
Arg$^{N\varepsilon}$	HB, SB	H-Asp31$^{O\delta2}$
Arg^{NH2}	HB, SB	H-Asp31$^{O\delta2}$
Arg^{N}	HB	H-Asp31^{O}
Arg^{O}	HB	H-Tyr97^{N}
**Asp794 (28.0)**		
Asp	VDW	H-Tyr96, H-Tyr97
**Ser795 (54.8)**		
Ser	VDW	H-Tyr96, H-Asp100B
**Asn797 (9.3)**		
Asn	VDW	H-Tyr32
Asn$^{O\delta1}$	HB	H-Tyr32^{OH}
**Val800 (13.6)**		
Val	VDW	H-Tyr97
**Phe801 (3.2)**		
Phe	VDW	H-Tyr97, H-Tyr98
**Asn802 (42.4)**		
Asn	VDW	H-Tyr33, H-Tyr97, H-Tyr98
Asn$^{O\delta1}$	HB	H-Tyr98^{N}
**Asn803 (31.4)**		
Asn	VDW	H-Tyr97, H-Tyr98, H-Gly99
Asn^{N}	HB	H-Tyr98^{O}
**Leu806 (69.1)**		
Leu	VDW	K-Trp91<br>H-Tyr98, H-Gly99
**Val807 (16.3)**		
Val	VDW	H-Tyr98
**Tyr820 (17.2)**		
Tyr	VDW	H-Tyr97

^5B3 residues are labelled according to the Kabat numbering system

Supplementary references

1. Scally SW, Triglia T, Evelyn C, et al. PCRCR complex is essential for invasion of human erythrocytes by *Plasmodium falciparum*. *Nature Microbiology*. 2022/12/01 2022;7(12):2039-2053. doi:10.1038/s41564-022-01261-2

2. Healer J, Wong W, Thompson JK, et al. Neutralising antibodies block the function of Rh5/Ripr/CyRPA complex during invasion of *Plasmodium falciparum* into human erythrocytes. *Cellular Microbiology*. 2019;21(7):e13030. doi:10.1111/cmi.13030
