# Supplementary material for: PTRAMP, CSS and Ripr form a conserved complex required for merozoite invasion of Plasmodium species into erythrocytes
Source: Nat Commun. 2026 Jan 26;17:1780. doi: 10.1038/s41467-026-68486-1 (PMC12916814; doi:10.1038/s41467-026-68486-1)
Supplement: Supplementary file 2 — Reporting summary [file 41467_2026_68486_MOESM2_ESM.pdf]

nature portfolio

Corresponding author(s): Alan F. Cowman
Stephen W. Scally

Last updated by author(s): Dec 18, 2025

Reporting Summary

Nature Portfolio wishes to improve the reproducibility of the work that we publish. This form provides structure for consistency and transparency in reporting. For further information on Nature Portfolio policies, see our Editorial Policies and the Editorial Policy Checklist.

Statistics

For all statistical analyses, confirm that the following items are present in the figure legend, table legend, main text, or Methods section.

n/a	Confirmed	
☐	☒	The exact sample size (*n*) for each experimental group/condition, given as a discrete number and unit of measurement
☐	☒	A statement on whether measurements were taken from distinct samples or whether the same sample was measured repeatedly
☒	☐	The statistical test(s) used AND whether they are one- or two-sided<br>*Only common tests should be described solely by name; describe more complex techniques in the Methods section.*
☒	☐	A description of all covariates tested
☒	☐	A description of any assumptions or corrections, such as tests of normality and adjustment for multiple comparisons
☐	☒	A full description of the statistical parameters including central tendency (e.g. means) or other basic estimates (e.g. regression coefficient) AND variation (e.g. standard deviation) or associated estimates of uncertainty (e.g. confidence intervals)
☒	☐	For null hypothesis testing, the test statistic (e.g. *F*, *t*, *r*) with confidence intervals, effect sizes, degrees of freedom and *P* value noted<br>*Give P values as exact values whenever suitable.*
☒	☐	For Bayesian analysis, information on the choice of priors and Markov chain Monte Carlo settings
☒	☐	For hierarchical and complex designs, identification of the appropriate level for tests and full reporting of outcomes
☒	☐	Estimates of effect sizes (e.g. Cohen's *d*, Pearson's *r*), indicating how they were calculated

Our web collection on statistics for biologists contains articles on many of the points above.

Software and code

Policy information about availability of computer code

Data collection	Cryo-EM data collection was carried out using EPU 3.8(Thermo Fisher)<br>Flow cytometry data was acquired using the Attune NxT software<br>Serology data was collected using Xponent software V.4.2(Thermofisher scientific)<br>BLI data was collected using Sartorius Octet Analysis studio v11.0

Data analysis	Sartorius Data Analysis (11.0)<br>XDS (v Jan 26, 2018)<br>Aimless (0.7.4)<br>Phaser (2.8.3)<br>Phenix (1.20.1)<br>Coot (0.9.8.3)<br>FlowJo (10.7)<br>ChimeraX (1.2-1.8)<br>Pymol (2.0)<br>CryoSPARC (4.4.1-4.6.2)<br>DiscoverMP (V 2023 R1)<br>ESPript (3.0)<br>GraphPad Prism (v9- 10)<br>AlphaFold 3<br>R (3.6.1)<br>R Studio (2024.12.1)

For manuscripts utilizing custom algorithms or software that are central to the research but not yet described in published literature, software must be made available to editors and reviewers. We strongly encourage code deposition in a community repository (e.g. GitHub). See the Nature Portfolio guidelines for submitting code & software for further information.

Data

Policy information about availability of data

All manuscripts must include a data availability statement. This statement should provide the following information, where applicable:

- Accession codes, unique identifiers, or web links for publicly available datasets
- A description of any restrictions on data availability
- For clinical datasets or third party data, please ensure that the statement adheres to our policy

The crystal structures reported in this manuscript have been deposited in the Protein Data Bank, www.rcsb.org (PDB ID codes 9NSD and 9YIO).

Research involving human participants, their data, or biological material

Policy information about studies with human participants or human data. See also policy information about sex, gender (identity/presentation), and sexual orientation and race, ethnicity and racism.

Reporting on sex and gender	In this study we utilised sets of existing human samples that were available from prior clinical trial or cohort studies. We therefore were not able to obtain any data that was not asked during these original studies. These studies requested patient/ participant sex, which we are referring to as self-reported sex as it was not biologically confirmed. Our patient samples are skewed towards men, due to higher risk of clinical infection in this group. Pf = 30 samples – 6 females, 24 males. Pk = 34 samples – 12 females, 22 males. Pv = 34 samples – 14 females, 20 males. We observed no statistically significant differences in antibody levels to each individual protein based on self-reported sex, however our study design was not powered for this analysis and hence this has not been included in the results
Reporting on race, ethnicity, or other socially relevant groupings	Not applicable. This information was not specifically included when recruiting participants.
Population characteristics	Pf = 30 samples – 6 females, 24 males (6 – 58 age range)<br>Pk = 34 samples – 12 females, 22 males (7 – 71 age range)<br>Pv = 34 samples – 14 females, 20 males (3 – 60 age range)
Recruitment	P. vivax invasion assays: Cambodian patients were recruited through cross sectional surveys or in malaria clinics and offered participation in the studies (supply of parasite-infected blood sample) after the study aims and protocol have been described. Written informed consent was obtained from all. Participation in the study did not affect treatment practices; malaria treatment was not provided by the research team, but by the clinical staff of the malaria outposts.<br><br>Serology: No recruitment was performed as part of this present study. Samples used were historical samples collected at various times. Details of recruitment can be found in the following publications:<br>Grigg et al Malaria Journal 2018<br>Longley et al J Trop Med Hyg 2016<br>Grigg et al Clin Infect Dis 2018<br>Longley et al Cell Rep 2022
Ethics oversight	WEHI Human Research Ethics Committee<br>National Ethics Committee for Health Research in Cambodia (192NECHR, July 11, 2022)<br>Faculty of Tropical Medicine, Mahidol University (MUTM 2014-025-01 and 02)<br>Menzies School of Health Research HREC (for Malaysian studies)(HREC 12-1815, 16-2544, 10-1431, 12-1807)

Note that full information on the approval of the study protocol must also be provided in the manuscript.

Field-specific reporting

Please select the one below that is the best fit for your research. If you are not sure, read the appropriate sections before making your selection.

☒ Life sciences ☐ Behavioural & social sciences ☐ Ecological, evolutionary & environmental sciences

For a reference copy of the document with all sections, see nature.com/documents/nr-reporting-summary-flat.pdf

Life sciences study design

All studies must disclose on these points even when the disclosure is negative.

Sample size	No statistical method was used to determine sample size.<br>Patients infected with P. vivax in Thailand (n = 34)<br>Patients infected with P. falciparum from Malaysia (n = 31) and P. knowlesi (n = 33)<br>Malaria naive individuals from Melbourne Volunteer Biospecimen Donor Registry (n = 28)<br>Malaria naive individuals from Thai Red Cross, Thailand (n = 29)<br>Afebrile healthy controls from Malaysia (n = 30)
Data exclusions	Afebrile healthy controls (n=30) were assayed from Sabah, Malaysia; however, these individuals may have had prior Plasmodium infections and were thus not utilized to create the seropositivity cut-off
Replication	Invasion assays were performed at least three times, with P. vivax assays being performed six times due to variability in the assay. Protein-protein interactions and protein-antibody BLI kinetic assays were performed at least two times. Nanobody kinetics BLI assays were performed once for each species. Mass photometry was performed once with technical replication. Flow cytometry was performed three times with independent blood samples. Analytical size-exclusion was repeated twice
Randomization	Randomization was not relevant to this study and best judgement was used to analyze data.
Blinding	No blinding was used in any data collection or data analysis.

Reporting for specific materials, systems and methods

We require information from authors about some types of materials, experimental systems and methods used in many studies. Here, indicate whether each material, system or method listed is relevant to your study. If you are not sure if a list item applies to your research, read the appropriate section before selecting a response.

Materials & experimental systems

n/a	Involved in the study	
☐	☒	Antibodies
☐	☒	Eukaryotic cell lines
☒	☐	Palaeontology and archaeology
☐	☒	Animals and other organisms
☒	☐	Clinical data
☒	☐	Dual use research of concern
☒	☐	Plants

Methods

n/a	Involved in the study	
☒	☐	ChIP-seq
☐	☒	Flow cytometry
☒	☐	MRI-based neuroimaging

Antibodies

Antibodies used	Anti-PvPTRAMP-CSS: 2D9, 4E2, 4H10, 5B3, 5B4, 5E11, 6A10<br>anti-PvPTRAMP-CSS nanobodies: A6, A7, B6, C6, C9, D4, D7, D8<br>Anti-PfPTRAMP-CSS nanobody: H2<br>anti-DARC antibody: 2C3<br>anti-PfRipr: 1G12<br>anti-PfRh5: 5A9<br>anti-PvRBP2b: rabbit pAb R1531<br>goat anti-mouse 488 (cat. no. A-11001, lot no. 2284614)<br>goat anti-mouse 647 (cat. no. A-21235, lot no. 2482945)<br>chicken anti-rabbit 647 (cat. no. A-21443, lot no. 2432061)<br>anti-tetanus toxin 043038<br>PE-conjugated anti-human secondary antibody (Jackson Immunoresearch)(product code 709-116-098)
Validation	goat anti-mouse 488 (cat. no. A-11001) validated by the supplier by IFA and flow cytometry<br>goat anti-mouse 647 (cat. no. A-21235) validated by the supplier by IFA and FACS<br>chicken anti-rabbit 647 (cat. no. A-21443) validated by the supplier by IFA and western blot<br>anti-PfRipr: 1G12 was validated in Healer et al. Cell Microbiol 2019 by western blot, SPR and GIA and in Scally et al. Nature Micro

2022 by GIA
anti-PfPTRAMP-CSS nanobody H2 was validated in Scally et al Nature Micro 2022 by BLI and GIA
anti-PfRh5: 5A9 was validated in Chen et al. PLOS Path 2011 by western blot and in Scally et al. Nature Micro 2022 by flow cytometry
anti-PvRBP2b: rabbit pAb R1531 validated in this paper in Extended data figure 10.
anti-tetanus toxin 043038 was validated in Carias et al. J Immunol 2019 and Popvici et al Nature Comms 2020
anti-DARC antibody: 2C3 was validated in Wasniowska et al. Transfusion Medicine 2002 and Popovici et al Nature Comms 2020
Anti-PvPTRAMP-CSS antibodies and nanobodies were validated in this paper by BLI, shown in Extended Data Figures 7 and 8 and supplementary material figures 2 and 5
PE-conjugated anti-human secondary antibody (Jackson Immunoresearch)(product code 709-116-098) was validated by the supplier by ELISA

Eukaryotic cell lines

Policy information about cell lines and Sex and Gender in Research

Cell line source(s)	YH1 P. knowlesi parasites<br>Berok R9 P. cynomolgi parasites<br>3D7 P. falciparum parasites<br>Expi293F cells (Thermofisher scientific)<br>Sf21 cells (Thermofisher scientific)<br>O+ erythrocyte (Australian Red Cross bloodbank, Melbourne, Australia)<br>Cord blood reticulocytes (Bone Marrow Donor Institute Cord Blood Bank, Royal Children's Hospital Melbourne, Australia)
Authentication	Parasite lines were periodically sequenced and were as expected. Sf21 and Expi293F cell lines were purchased and included the certificate of analysis from the supplier.
Mycoplasma contamination	All cell lines were tested periodically for Mycoplasma contamination and were negative.
Commonly misidentified lines<br>(See ICLAC register)	N/A

Animals and other research organisms

Policy information about studies involving animals; ARRIVE guidelines recommended for reporting animal research, and Sex and Gender in Research

Laboratory animals	Two female Balb/C mice received immunisation at 8-9 weeks of age. Animals were housed in open top cages with autoclaved bedding, and were checked daily. Dark/light cycle 12hrs - 7pm-7am dark - 7am-7pm light. Temperature was set to 21C.<br><br>One female alpaca was immunised with PvCSS and PvPTRAMP for the generation of nanobodies.
Wild animals	no wild animals were used in this study
Reporting on sex	all animals used were female. No analysis was performed as animals were used only for antibody/nanobody production
Field-collected samples	no field samples were used in this study
Ethics oversight	Antibodies were raised in mice and according to procedures approved by the Walter and Eliza Hall Institute of Medical Research Animal Ethics Committee. Alpaca handling and immunisation was approved by Agriculture Victoria, Wildlife and Small Institutions Animal Ethics Committee, project approval No. 26-17

Note that full information on the approval of the study protocol must also be provided in the manuscript.

Plants

Seed stocks	no plants were used in this study
Novel plant genotypes	N/A
Authentication	N/A

Flow Cytometry

Plots

Confirm that:

☒ The axis labels state the marker and fluorochrome used (e.g. CD4-FITC).

☒ The axis scales are clearly visible. Include numbers along axes only for bottom left plot of group (a 'group' is an analysis of identical markers).

☒ All plots are contour plots with outliers or pseudocolor plots.

☒ A numerical value for number of cells or percentage (with statistics) is provided.

Methodology

Sample preparation	For assays using mature erythrocytes, erythrocytes were washed twice in PBS and then made up to a density of approximately 1 x 107 cells/mL in PBS + 1% (w/v) BSA (PBS-BSA). Each sample used 100 µL of this suspension. Erythrocytes were centrifuged, the supernatant was removed, and the cells were resuspended in a solution containing freshly prepared recombinant proteins in PBS- BSA. Individual proteins were prepared at a final concentration of 2 µM (except for PfRh5, which was prepared at 400 nM), and complexes were mixed with an equimolar amount of protein to a final concentration of 2 µM. After a 45-minute incubation at room temperature, the samples were centrifuged, washed, and then incubated with primary antibodies, either 5A9 (anti-Rh5), 4E2 (anti-PC) or 5E11 (anti-Ripr). After a 45-minute incubation the cells were again centrifuged and then incubated with Alexa-488 anti-mouse fluorescent antibody at a dilution of 1:100. After a 45- minute incubation, cells were washed twice in PBS and then resuspended before analysis on an Attune NxT flow cytometer (Thermo Fisher Scientific). For each sample 50,000 events were recorded.<br><br>For assays involving reticulocyte-enriched cord blood, erythrocytes were made up in 1x HTPBS + 1% (w/v) BSA (HTPBS-BSA) to a density of approximately 1 x 107 cells/mL. Each sample used 100 µL of this suspension. Reticulocytes were centrifuged (2000 x g for one minute), the HTPBS- BSA removed, and then resuspended in a solution containing recombinant proteins in HTPBS- BSA and incubated at room temperature for 45 minutes. PvPC and PkPC were used at a final concentration of 2 µM. Samples were centrifuged after which the protein solution removed, and cells were washed once with HTPBS-BSA and then incubated with 4E2 (anti-PC) at a concentration of 0.05 mg/mL or polyclonal sera (anti-RBP2b) at a concentration of 12.5 µg/mL. After a 45-minute incubation, the cells were again centrifuged, and the antibody solution was removed. Cells were washed once as before and then stained with Alexa-647 (either anti-rabbit or anti-mouse) at a dilution of 1:100. After 45 minutes the reticulocytes were again washed and incubated with 50 µL of thiazole orange (BD Retic-Count, BD Biosciences) for 30 minutes. Finally, the reticulocytes were centrifuged, the Retic-Count solution removed, and cells were washed with 1x HTPBS two times before analysis on an Attune NxT flow cytometer (Thermo Fisher Scientific). For each sample 50,000 events were recorded. The data were then analysed in FlowJoTM v10.7 Software (BD Life Sciences). This involved gating reticulocytes and then applying a quadrant gate according to the thiazole orange staining and the background staining of the antibody in combination with the Alexa 647. This antibody background was subtracted from the positive population recorded in the presence of recombinant protein: this background-subtracted value has been plotted in the summary figures. Positive binding is determined by the double positive population in the upper right-hand quadrant.<br><br>Parasitemia was determined using flow cytometry (BD Acurri) after staining with 10 mg/mL of ethidium bromide, with data analysed using FlowJo software (BD Life Sciences).
Instrument	Attune NxT, BD Acurri
Software	Attune NxT software. FlowJoTM v10.7
Cell population abundance	Relevant cell proportions are displayed in Supplementary Figure 16 and were not assessed further or sorted
Gating strategy	Erythrocytes were gated SSC-A and FSC-A, then doublets were excluded using FSC-H and FSC-A, binding was determined as alexa-488 positive cells. For reticulocyte binding, a similar approach was used and binding determined by double-positive events (alexa 647 positive and 488nm (thiazole) positive). An example of the gating strategy used is shown in Supplementary Figure 16.

☒ Tick this box to confirm that a figure exemplifying the gating strategy is provided in the Supplementary Information.
